# General Anesthesia vs. Local Anesthesia During Endovascular Treatment for Acute Large Vessel Occlusion: A Propensity Score-Matched Analysis

**DOI:** 10.3389/fneur.2021.801024

**Published:** 2022-02-14

**Authors:** Hongxing Han, Yu Wang, Hao Wang, Hongyang Sun, Xianjun Wang, Jian Gong, Xiaochuan Huo, Qiyi Zhu, Fengyuan Che

**Affiliations:** ^1^Department of Neurology, Linyi People's Hospital, Linyi, China; ^2^Department of Interventional Neuroradiology, Beijing Tiantan Hospital, Capital Medical University, Beijing, China

**Keywords:** general anesthesia, local anesthesia, endovascular treatment, large vessel occlusion, propensity score matching

## Abstract

**Objective:**

To date, no consensus still exists on the anesthesia strategy of endovascular treatment (EVT) for acute ischemic stroke (AIS) due to large vessel occlusion (LVO). We aimed to compare the 90-day outcomes, puncture-to-recanalization time (PRT), successful recanalization rate, and symptomatic intracranial hemorrhage (sICH) of patients undergoing general anesthesia (GA) or local anesthesia (LA) ± conscious sedation (CS) during the procedure.

**Methods:**

We selected patients from the Acute Ischemic Stroke Cooperation Group of Endovascular Treatment (ANGEL) registry and divided them into the GA group and the LA ± CS group. The two groups underwent 1:1 matching under propensity score matching (PSM) analysis. Then, we compared the primary outcome including the 90-day modified Rankin Scale (mRS) 0–2, secondary outcome including the 90-day mRS, the 90-day mRS 0–1, the 90-day mRS 0–3, PRT, and successful recanalization rate as well as the safety outcome including sICH, any ICH, and 90-day mRS 6.

**Results:**

Among the 705 enrolled patients, 263 patients underwent GA and 442 patients underwent LA ± CS. After 1:1 PSM according to the baseline characteristics, each group has 216 patients. Patients with GA had the higher median 90-day mRS [3 (1–5) vs. 2 (1–4), *p* < 0.001], the lower 90-day mRS 0–2 rate (43.5 vs. 56.5%, *p* = 0.007), higher mortality (19.9 vs.10.2%, *p* = 0.005), and longer PRT [92 (60–140) vs. 70 (45–103) min, *p* < 0.001]. There were no differences in sICH and successful recanalization rate between both the groups.

**Conclusion:**

In the real-world setting, LA ± CS might provide more outcomes benefits than GA in patients with AIS-LVO during the procedure.

## Introduction

Endovascular treatment (EVT) has become the standard for acute ischemic stroke (AIS) due to large vessel occlusion (LVO) (1–5). However, the most suitable anesthetic approach is still unknown. Recently, three well-known randomized controlled trials (RCTs) [Sedation vs Intubation for Endovascular Stroke Treatment (SIESTA), General or Local Anesthesia in Intra Arterial Therapy (GOLIATH), and Anesthesia During Stroke (ANSTROKE)] showed no significant difference in the outcome between different anesthetic approaches (6–8). Surprisingly, a meta-analysis of these three trials demonstrated different results; the use of protocol-based general anesthesia (GA) was significantly associated with less disability at 3 months (9). Conversely, analysis from the Highly Effective Reperfusion Evaluated in Multiple Endovascular Stroke Trials (HERMES) collaborators demonstrated an association between poor outcome and GA (10). The finding from the Endovascular Therapy Following Imaging Evaluation for Ischemic Stroke (DEFUSE 3) trial *post-hoc* analysis supported this result (11).

In a real-world scenario, the result might be different. Hence, the objective of this study was to compare the safety and efficacy outcomes between different anesthetic approaches, mainly GA vs. local anesthesia (LA) ± conscious sedation (CS) in patients with AIS-LVO undergoing EVT using data from the prospective multicenter Acute Ischemic Stroke Cooperation Group of Endovascular Treatment (ANGEL) registry.

## Methods

### Patient Population and Data Collection

We retrospectively reviewed patients from a multicenter, prospective study of the ANGEL registry from June 2015 to December 2017 (12). Inclusion criteria in this study were described as the following: (1) Age more than 18 years; (2) Clinical diagnosis of ischemic stroke in which the stroke symptoms last for more than 30 min and no improvement prior to treatment; (3) The modified Rankin Scale (mRS) less than 2 before the current stroke; (4) Large vessel occlusion in the internal carotid artery (ICA), middle cerebral artery (MCA) (M1/M2 segment), and anterior cerebral artery (ACA); and (5) Informed consent form was obtained from the patient or legally authorized representative of the patient after receiving information about data collection.

Of all the patients, we excluded 210 patients due to posterior circulation stroke (*n* = 203) and no thrombectomy procedure [only digital subtraction angiography (DSA) or fragment, *n* = 7]. Finally, we classified 705 patients with GA (*n* = 263) and LA ± CS (*n* = 442). There were 61 (8.7%) patients who received CS in this study. GA was defined as induction and maintenance with sedation drugs, analgesic agents, and muscle relaxants, with controlled ventilation under tracheal intubation or laryngeal mask, from the time of puncture to the end of the procedure. CS was defined as LA and spontaneous breathing, with administration of sedatives during the procedure. LA is defined as subcutaneous anesthesia at the arterial puncture site with or without administration of sedatives throughout the procedure (13).

We recorded the demographics, medical history, prior treatment [antiplatelet therapy and intravenous thrombolysis (IVT)], systolic blood pressure (SBP), the National Institutes of Health Stroke Scale (NIHSS) score, the Alberta Stroke Program Early CT Score (ASPECTS) (14), occlusion sites [ICA, MCA (M1, M2/M3), ACA, and tandem occlusion] (15), the Trial of ORG 10172 in Acute Stroke Treatment (TOAST) stroke subtypes (16), procedural characteristics, and the time points of working flow of the patient. All the pretreatment imaging data, including noncontrast CT, MRI, and DSA images during EVT and follow-up CT or MRI of the head, were anonymized and reviewed centrally by two independent physicians. A consensus between the physicians was obtained to resolve any disagreements; if no agreement was achieved, then a third physician blinded to this study was introduced for a final consensus.

### Outcomes

The primary functional outcome was the 90-day mRS 0–2. Meanwhile, the safety endpoints were symptomatic intracranial hemorrhage (sICH) within 24 h post hours, which was diagnosed according to the European Cooperative Acute Stroke Study (ECASS-II) (17), any ICH within 24 h post-EVT, and mortality (mRS 6). Secondary outcomes included the 90-day mRS, the 90-day mRS 0–1, the 90-day mRS 0–3, successful recanalization of the modified Tissue Thrombolysis in Cerebral Ischemia (mTICI) 2b/3, and time from puncture to recanalization (18). At 3 months after endovascular therapy, we assessed the prognoses of all the patients through telephone follow-up. The follow-up was based on a shared standardized interview protocol and centrally conducted by a third-party Clinical Research Organization (CRO) blinded to the clinical details or anesthesia method.

### Statistical Analysis

We described the categorical variables as numbers and percentages. We expressed the continuous variables as median with [interquartile range (IQR)]. We use the Wilcoxon rank-sum test for continuous variables and the Pearson's chi-squared test or the Fisher's exact test for categorical variables to perform univariate analysis to find the different characteristics between the GA and LA groups. Then, we performed propensity score matching (PSM) analysis using the caliper size of 0.02 to reduce selection bias and confounding variables between the two groups at a 1:1 ratio. All the significant baseline characteristics in univariate analysis (*p* < 0.05) and the baseline variables likely to influence the outcome were in the multivariate logistic regression model to calculate the propensity score including age, SBP, the NIHSS, the ASPECTS, IVT, and antiplatelet therapy before EVT, large artery atherosclerosis (LAA) stroke subtype, cardioembolism (CE) stroke subtype, tandem lesion, occlusion location, and time from door to puncture. Following the score generation, the neighboring matching algorithm without replacement was used to match the GA group and the LA ± CS group. After PSM, we used the same statistical methods to compare the two groups. A *p* < 0.05 (two-sided) was considered as statistically significant. We used the SPSS version 25.0 (IBM Incorporation, Armonk, New York, USA) to analyze the data.

## Results

**Figure 1:**
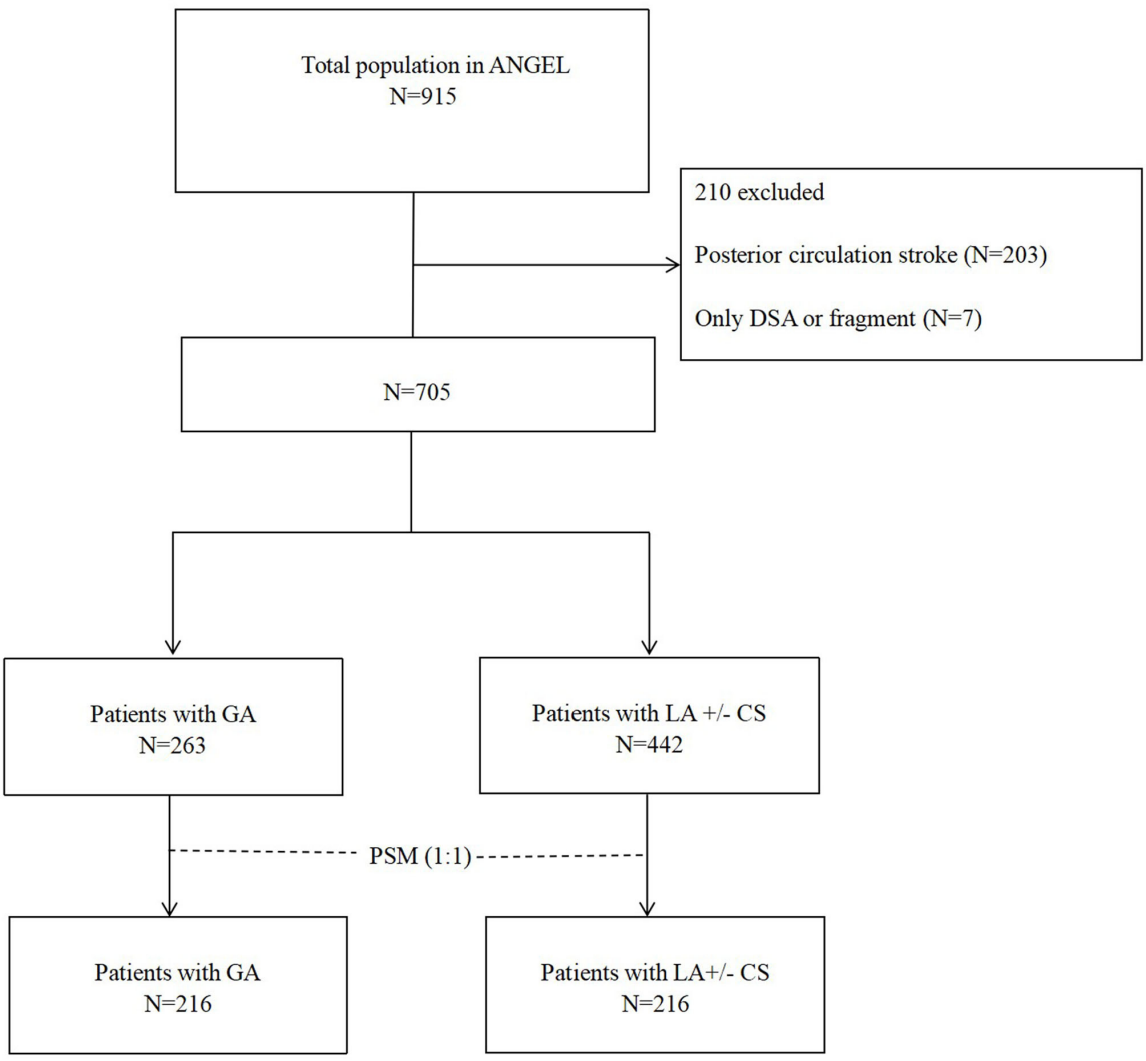
Flowchart of patient selection. PSM, propensity score matching; GA, general anesthesia; LA, local anesthesia; CS, conscious sedation; DSA, digital subtraction angiography.

Table 1 shows that 705 patients with AIS in anterior circulation underwent EVT were included in this study. During EVT, 263 patients received GA and 442 patients received LA (Figure 1). Antiplatelet therapy was significantly different before EVT, IVT before EVT, the admission NIHSS, the admission ASPECTS, tandem occlusion, LAA subtype, CE subtype, and time from door to puncture between the two groups. Compared to the LA ± CS group, patients in the GA group had the higher median NIHSS, the longer median time from door to puncture, and the median ASPECTS. Besides antiplatelet therapy and CE subtype, IVT before EVT, LAA subtype, and tandem occlusion occurred less frequently in the GA group.

**Table 1:** Comparison of baseline, procedure, and outcome characteristics between the two groups before PSM.

**Variables/clinical findings**	**All Patients** **(***n*** = 705)**	**GA** **(***n*** = 263)**	**LA ±CS** **(***n*** = 442)**	***P*** **-value**
Age, y, median (IQR)	64 (55–73)	65 (58–73)	64 (55–73)	0.170
Men, *n* (%)	495 (64.5)	165 (62.7)	290 (65.6)	0.441
**Medical history**, ***n*** **(%)**				
Hypertension	364 (51.6)	131 (49.8)	233 (52.7)	0.455
Diabetes mellitus	107 (15.2)	35 (13.3)	72 (16.3)	0.286
Atrial fibrillation	145 (20.6)	57 (21.7)	88 (19.9)	0.575
Stroke	72 (10.2)	25 (9.5)	47 (10.6)	0.632
Current smoking	237 (33.6)	87 (33.1)	150 (33.9)	0.816
Current drinking	101 (14.3)	40 (15.2)	61 (13.8)	0.606
**Prior treatment**, ***n*** **(%)**				
Antiplatelet therapy	150 (21.3)	72 (27.4)	78 (17.6)	**0.002**
IVT	263 (37.3)	63 (24.0)	143 (32.4)	**0.018**
**Baseline measurements**				
SBP, mmHg	145 (130–161)	147 (130–164)	144 (130–160)	0.287
Admission NIHSS, median (IQR)	15 (10–20)	17 (13–21)	13 (9–18)	**<0.001**
ASPECTS, median (IQR)	8 (7–8)	8 (7–8)	8 (7–8)	**0.024**
**Occlusion site**, ***n*** **(%) 0.093**				
ICA	285 (40.4)	113 (43.0)	172 (38.9)	
M1	333 (47.2)	128 (48.7)	205 (46.4)	
M2/3	82 (11.6)	21 (8.0)	61 (13.8)	
ACA	5 (0.7)	1 (0.4)	4 (0.9)	
Tandem occlusion	126 (17.9)	35 (13.3)	91 (20.6)	**0.015**
**Stroke subtype**, ***n*** **(%)**				
LAA	486 (68.9)	164 (62.4)	322 (72.9)	**0.004**
CE	144 (20.4)	71 (27.0)	73 (16.5)	**0.001**
**Procedure process**, ***n*** **(%)**				
GP IIb/IIIa receptor inhibitor	213 (30.2)	106 (40.3)	107 (24.2)	**<0.001**
Stent retriever	528 (74.9)	237 (90.1)	291 (65.8)	**<0.001**
Aspiration	51 (7.2)	24 (9.1)	27 (6.1)	0.135
IAT	230 (32.6)	82 (31.2)	148 (33.5)	0.528
Angioplasty	53 (7.5)	18 (6.8)	35 (7.9)	0.601
Stenting	98 (13.9)	43 (16.3)	55 (12.4)	0.147
**Time intervals, min, median (IQR)**				
Onset-to-door time	180 (95–270)	180 (105–255)	180 (90–278.5)	0.642
Door-to-puncture time	110 (69.5–156)	115 (74–165)	105 (57–140)	**0.007**
Onset-to-recanalization time	385 (300–505)	393.5 (301.75–487.25)	380 (296–510)	0.655
**Primary outcome**, ***n*** **(%)**				
90-day mRS 0–2	369 (52.3)	107 (40.7)	262 (59.3)	**<0.001**
**Secondary outcomes**				
90-day mRS, median (IQR)	3 (1–4)	3 (1–5)	2 (1–4)	**<0.001**
90-day mRS 0–1, *n* (%)	292 (41.4)	82 (31.2)	210 (47.5)	**<0.001**
90-day mRS 0–3, *n* (%)	465 (66.0)	146 (55.5)	319 (72.2)	**<0.001**
Puncture-to-recanalization time, median (IQR)	80 (50–112)	89.5 (60–140)	75 (50–110)	**<0.001**
Successful recanalization (mTICI 2b/3), *n* (%)	649 (92.1)	244 (92.8)	405 (91.6)	0.586
**Safety outcomes**, ***n*** **(%)**				
sICH	44 (6.2)	22 (8.4)	22 (5.0)	0.072
Any ICH	166 (23.5)	69 (26.2)	97 (21.9)	0.194
90-day mRS 6	110 (15.6)	56 (21.3)	54 (12.2)	**0.001**

*PSM, propensity score matching; IQR, interquartile range; IVT, intravenous thrombolysis; NIHSS, National Institutes of Health Stroke Scale; ASPECTS, Alberta Stroke Program Early CT Score; ICA, internal carotid artery; M1, middle cerebral artery M1 segment; M2, middle cerebral artery M2 segment; M3, middle cerebral artery M3 segment; ACA, anterior cerebral artery; SBP, systolic blood pressure; LAA, large artery atherosclerosis; CE, cardioembolism; IAT, intra-arterial thrombolysis; ICH, intracranial hemorrhage; sICH, symptomatic ICH; mRS, modified Rankin Scale; GA, general anesthesia; LA, local anesthesia, CS, conscious sedation. Bold values indicates statistical significance*.

Table 2 shows that 216 patients in each group were matched 1:1 according to the baseline characteristics. After PSM, the covariates were statistically similar between the two groups. The proportion of patients receiving antiplatelet therapy (25.5 vs. 28.2%, *p* = 0.515) and IVT (28.2 vs. 29.6%, *p* = 0.750) before EVT in the GA group was similar to the LA ± CS group. There was no significant difference in tandem occlusion (14.8 vs. 13.9%, *p* = 0.784), LAA subtype (67.1 vs. 47.3%, *p* = 0.134) and CE subtype (23.1 vs. 23.1%, *p* = 1.000) between the two groups. The admission NIHSS (16 vs. 16, *p* = 0.589), the ASPECTS (8 vs. 8, *p* = 0.523), and time from door to puncture (110 vs. 104.5 min, *p* = 0.669) were similar between the two groups.

**Table 2:** Comparison of baseline, procedure, and outcome characteristics between the two groups after PSM.

**Variables/clinical findings**	**All patients** **(***n*** = 432)**	**GA** **(***n*** = 216)**	**LA ±CS** **(***n*** = 216)**	***P*** **-value**
Age,y, median (IQR)	64 (55–73)	64 (56–73)	63 (54–74.8)	0.472
Men, *n* (%)	277 (64.1)	137 (63.4)	140 (64.8)	0.763
**Medical history**, ***n*** **(%)**				
Hypertension	217 (105)	105 (48.6)	112 (51.9)	0.501
Diabetes mellitus	69 (16.0)	31 (14.4)	38 (17.6)	0.358
Atrial fibrillation	92 (21.3)	41 (19.0)	51 (23.6)	0.240
Stroke	44 (10.2)	20 (9.3)	24 (11.1)	0.525
Current smoking	155 (35.9)	78 (36.1)	77 (35.6)	0.920
Current drinking	62 (14.4)	36 (16.7)	26 (12.0)	0.170
**Prior treatment**, ***n*** **(%)**				
Antiplatelet therapy	116 (26.9)	55 (25.5)	61 (28.2)	0.515
IV thrombolysis	125 (28.9)	61 (28.2)	64 (29.6)	0.750
**Baseline measurements, median (IQR)**				
Systolic blood pressure, mmHg	146 (130–162)	148.5 (130.0–163.5)	145.5 (130.0–162.0)	0.738
Admission NIHSS	16 (13–21)	16 (13–21)	16 (13–22)	0.589
ASPECTS, median	8 (7–8)	8 (7–8)	8 (7–8)	0.523
**Occlusion site**, ***n*** **(%)**				
ICA	174 (40.3)	94 (43.5)	80 (37.0)	0.163
M1	205 (47.5)	102 (47.2)	103 (47.7)	
M2/3	51 (11.8)	19 (8.8)	32 (14.8)	
ACA	2 (0.5)	1 (0.5)	1 (0.5)	
Tandem occlusion	62 (14.4)	32 (14.8)	30 (13.9)	0.784
**TOAST**, ***n*** **(%)**				
LAA	275 (63.7)	145 (67.1)	130 (47.3)	0.134
CE	100 (23.1)	50 (23.1)	50 (23.1)	1.000
**Procedure process**, ***n*** **(%)**				
GP IIb/IIIa receptor inhibitor	147 (34.0)	83 (38.4)	64 (29.6)	0.054
Stent retriever	357 (82.6)	192 (88.9)	165 (76.4)	**0.001**
Aspiration	40 (9.3)	20 (9.3)	20 (9.3)	1.000
IA thromblysis	118 (27.3)	73 (33.8)	45 (20.8)	0.002
Angioplasty	40 (9.3)	18 (8.3)	22 (10.2)	0.507
Stenting	69 (16.0)	38 (17.6)	31 (14.4)	0.358
**Time intervals, min, median (IQR)**				
Onset-to-door time	160 (90–255)	170 (90.5–241.0)	154.0 (87.8–266.0)	0.510
Door-to-puncture time	106 (60.8–150)	110 (60–145)	104.5 (65.0–150.0)	0.669
Onset-to-recanalization time	374.5 (284.5–470)	397 (300–487)	360 (277–460)	**0.012**
**Primary outcome**, ***n*** **(%)**				
90-day mRS 0–2	216 (50.0)	94 (43.5)	122 (56.5)	**0.007**
**Secondary outcomes**				
90-day mRS, median (IQR)	3 (1–4)	3 (1–5)	2 (1–4)	**<0.001**
90-day mRS 0–1, *n* (%)	169 (39.1)	72 (33.3)	97 (44.9)	**0.014**
90-day mRS 0–3, *n* (%)	276 (63.9)	125 (57.9)	151 (69.9)	**0.009**
Puncture-to-recanalization time, median (IQR)	80 (50–115)	92 (60–140)	70 (45–103)	**<0.001**
Successful recanalization (mTICI 2b/3), *n* (%)	390 (90.3)	200 (92.6)	190 (88.0)	0.104
**Safety outcomes**, ***n*** **(%)**				
Any ICH	112 (25.9)	56 (25.9)	56 (25.9)	1.000
sICH	28 (6.5)	17 (7.9)	11 (5.1)	0.241
90-day mRS 6	65 (15.0)	43 (19.9)	22 (10.2)	**0.005**

*PSM, propensity score matching; IQR, interquartile range; IVT, intravenous thrombolysis; NIHSS, National Institutes of Health Stroke Scale; ASPECTS, Alberta Stroke Program Early CT Score; ICA, internal carotid artery; M1, middle cerebral artery M1 segment; M2, middle cerebral artery M2 segment; M3, middle cerebral artery M3 segment; ACA, anterior cerebral artery; SBP, systolic blood pressure; LAA, large artery atherosclerosis; CE, cardioembolism; IAT, intra-arterial thrombolysis; ICH, intracranial hemorrhage; sICH, symptomatic ICH; mRS, modified Rankin Scale; GA, general anesthesia; LA, local anesthesia, CS, conscious sedation. Bold values indicates statistical significance*.

**Figure 2:**
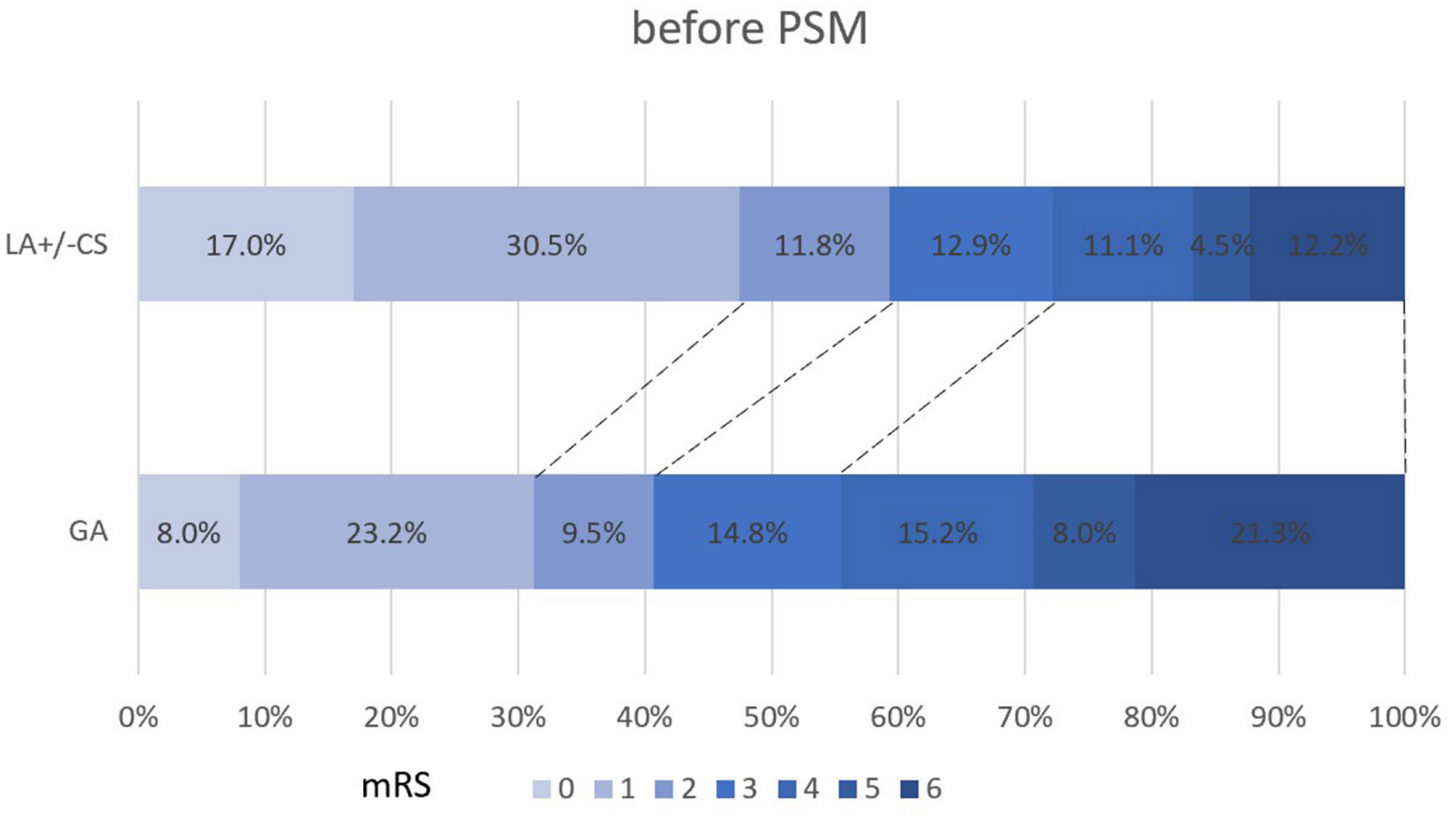
Shift on the 90-day mRS score stratified by LA and GA before PSM. PSM, propensity score matching; mRS, modified Rankin Scale.

**Figure 3:**
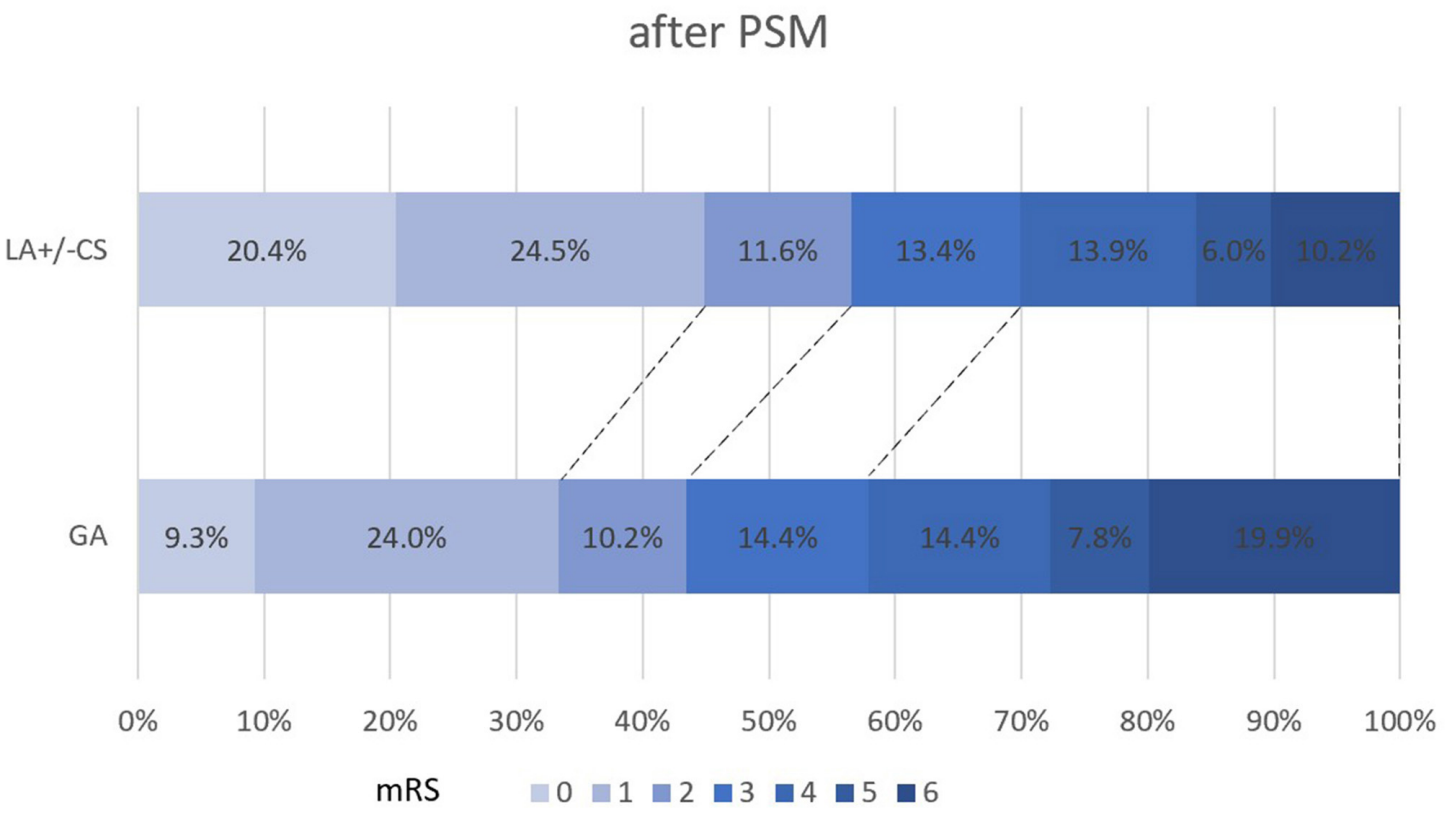
Shift on the 90-day mRS score stratified by LA ± CS and GA after PSM. PSM, propensity score matching; mRS, modified Rankin Scale; GA, general anesthesia; LA, local anesthesia; CS, conscious sedation.

The propensity score-adjusted outcomes of the two groups are shown in Table 2. Time from puncture to recanalization (92 vs. 70 min, *p* = 0.000) and time from onset to recanalization (397 vs. 360 min, *p* = 0.012) were longer in the GA group than the LA ± CS group. The median mRS at 90 days was higher in the GA group than the LA ± CS group (3 vs. 2, *p* = 0.000). Compared to the LA ± CS group, excellent outcome rate at 90 days (33.3 vs. 44.9%, *p* = 0.014), good outcome rate at 90 days (43.5 vs. 56.5%, *p* = 0.007), and favorable outcome rate at 90 days (57.9 vs. 69.9%, *p* = 0.009) were lower, while mortality at 90 days (19.9 vs. 10.2%, *p* = 0.005) was higher in the GA group (Figures 2, 3). There was no difference in successful recanalization rate, any ICH incidence, and sICH incidence between the two groups.

## Discussion

In this multicenter study, in patients with AIS-LVO who had successful recanalization, LA may provide more functional benefit at 3 months follow-up than GA. A shorter time workflow from LA might attribute to this outcome result.

There is still inconsistency regarding the anesthetic approach during EVT. A study using PSM to reduce the impact of confounding factors reported that CS might reduce the in-hospital mortality, rates of complications, hospital costs, and lengths of stay than those who had GA (19). Another cohort study reported a similar result (20). In contrast, the Solitaire with the Intention for Thrombectomy as Primary Endovascular Treatment (SWIFT PRIME) investigators (21) demonstrated that GA has a comparable time to treatment initiation and successful revascularization. Although, their study also demonstrated the negative effect of GA such as lower rates of functional independence and an increase in periprocedural hypotension and postoperative pneumonia.

Later, three well-known RCTs demonstrated that anesthesia patterns might not significantly impact clinical treatment after EVT (7–9). However, the lack of sample size and centers involved limited the result to be generalized into the global population. Surprisingly, the meta-analysis of these three RCTs showed different results (9). GA is associated with clinical benefits in this study. The higher reperfusion rates in the GA group might attribute this result, suggesting that more optimal procedural condition during EVT is essential to the functional outcome.

On the contrary, a meta-analysis of individual data by the HERMES collaborators demonstrated that the non-GA approach showed better functional outcomes after EVT than those with the GA approach (10). Nevertheless, the largely unbalanced baseline parameters increase the risk for bias and confounding. Furthermore, the incomplete anesthesia pattern and hemodynamic management information further limited their interpretability.

Particular caution is needed when interpreting those results of studies. Either GA or non-GA both has advantages and disadvantages. GA may have the advantage in achieving a higher recanalization rate, as operators were more convenient to perform more complex EVT procedures confronting complex lesions in the presence of patient agitation and discomfort. Besides, the circumstances might lower the probability of procedural complications such as arterial perforation. These advantages might be less provided by non-GA (22). On the other hand, hypotension and blood pressure variability were more common in those who had GA, exacerbating the functional outcomes (9, 22).

While most studies compared the effect of GA and non-GA, the most non-GA approach was CS. To date, there is still a lack of study investigating the impact of LA in patients with AIS-LVO undergoing EVT. Recently, only two studies using PSM analysis investigated the impact of LA and non-LA on EVT outcomes (22, 23). Both studies demonstrated that non-LA was associated with better clinical outcomes. Nevertheless, the lower reperfusion rates in the LA ± CS group might influence this result. Inconsistent with that, LA demonstrated better functional outcomes at 90-day follow-up in this study. Despite similar reperfusion rates achieved in the LA and GA groups, LA showed a significantly shorter duration of time workflow. This result highlighted the importance of time workflow in modifying the outcome in different anesthesia approaches.

This study could not conclude which anesthesia approach is the best for EVT. However, we recommended that the procedure for anesthesia should be individualized according to the preoperative integrative assessment of the status of the patient. Non-GA, particularly LA, is first recommended. However, if the conditions were not allowed, there should be no argument for choosing GA. In the circumstances, attempts should be made to avoid the delay of the anesthesia procedure and hypotension to optimize the treatment outcomes.

This study has several limitations. First, the nonrandomized design. Despite using propensity score analysis to minimize the impact of confounding factors, other unmentioned factors may also impact the treatment outcome. Second, the small sample size and this study is limited to the Chinese population. Thus, this result could not be generalized to the global population. Third, LA is the first recommended anesthesia approach and GA was preferred in patients with more severe stroke. Therefore, there was a high probability of selection bias, which may affect the treatment result.

## Conclusion

Our multicenter study data suggest that LA ± CS could be superior to GA for those who achieved successful recanalization. Future trials are needed to determine the best anesthetic approach for AIS-LVO in different patient stratification.

## Data Availability Statement

The raw data supporting the conclusions of this article will be made available by the authors, without undue reservation.

## Ethics Statement

The studies involving human participants were reviewed and approved by Ethics Committee of Beijing Tiantan Hospital. The patients/participants provided their written informed consent to participate in this study.

## Author Contributions

FC and QZ supervised and performed quality control for the study. HH performed statistical analysis and acquired the data and wrote the manuscript with input from all the co-authors. All the authors contributed to the article and approved the submitted version of the manuscript.

## Funding

This study was supported by the National Key Research and Development Program of China, grant no. 2016YFC1301500.

## Conflict of Interest

The authors declare that the research was conducted in the absence of any commercial or financial relationships that could be construed as a potential conflict of interest.

## Publisher's Note

All claims expressed in this article are solely those of the authors and do not necessarily represent those of their affiliated organizations, or those of the publisher, the editors and the reviewers. Any product that may be evaluated in this article, or claim that may be made by its manufacturer, is not guaranteed or endorsed by the publisher.
